# Minirhizotron measurements can supplement deep soil coring to evaluate root growth of winter wheat when certain pitfalls are avoided

**DOI:** 10.1186/s13007-024-01313-0

**Published:** 2024-12-17

**Authors:** Jessica Arnhold, Facundo R. Ispizua Yamati, Henning Kage, Anne-Katrin Mahlein, Heinz-Josef Koch, Dennis Grunwald

**Affiliations:** 1 Institute of Sugar Beet Research, Holtenser Landstraße 77, 37079 Göttingen, Germany; 2 Institute of Crop Science and Plant Breeding, Kiel University, 24118 Kiel, Germany

**Keywords:** Root sampling, Root length estimation, Convolutional neural network, Root image segmentation, Soil core sampling

## Abstract

**Background:**

Root growth is most commonly determined with the destructive soil core method, which is very labor-intensive and destroys the plants at the sampling spots. The alternative minirhizotron technique allows for root growth observation throughout the growing season at the same spot but necessitates a high-throughput image analysis for being labor- and cost-efficient. In this study, wheat root development in agronomically varied situations was monitored with minirhizotrons over the growing period in two years, paralleled by destructive samplings at two dates. The aims of this study were to (i) adapt an existing CNN-based segmentation method for wheat minirhizotron images, (ii) verify the results of minirhizotron measurements with root growth data obtained by the destructive soil core method, and (iii) investigate the effect of the presence of the minirhizotron tubes on root growth.

**Results:**

The previously existing CNN could successfully be adapted for wheat root images. The minirhizotron technique seems to be more suitable for root growth observation in the subsoil, where a good agreement with destructively gathered data was found, while root length results in the topsoil were dissatisfactory in comparison to the soil core method in both years. The tube presence was found to affect root growth only if not installed with a good soil-tube contact which can be achieved by slurrying, i.e. filling gaps with a soil/water suspension.

**Conclusions:**

Overall, the minirhizotron technique in combination with high-throughput image analysis seems to be an alternative and valuable technique for suitable research questions in root research targeting the subsoil.

**Supplementary Information:**

The online version contains supplementary material available at 10.1186/s13007-024-01313-0.

## Background

Rooting depth and intensity significantly influence the capability of plants to take up water and nutrients, thus detailed knowledge on root dynamics during the vegetation period is required to understand the involved functional mechanisms. Regarding winter wheat, root growth is closely correlated with the above-ground biomass formation and grain yield as reported by [1] who extended a wheat crop model in simulation of root growth and plant development. Accordingly, [2] reported that wheat grain yield is strongly affected by the plants’ root growth, as investigated for different cultivation patterns of wheat in China. European wheat grain yields stagnate since the 1990s [3] which may be ameliorated by improving wheat root exploration of the soil. For this, efficient root analysis tools are required to support the selection of wheat genotypes with a deep root system and the ability to explore deep soil water and nutrient resources [4].

Historically, destructive methods like the soil core method were dominant in research on root growth [5, 6]. However, taking and processing soil cores is very labor-intensive, and when taking soil cores, plants at the sampling spot are destroyed and thus the sampled plants cannot be observed further throughout the growing season. Therefore, the comparatively less invasive minirhizotron method in combination with image analysis became more common over time [7–9]. The minirhizotron method can be used for continuous, non-destructive root growth observations, using a camera system to take belowground images from roots surrounding a transparent tube previously installed in the soil [8]. The tubes are expected to disturb the environment of the roots and the plant itself only minimally and allow an image acquisition at a high temporal resolution [9, 10]. Images may be analyzed manually, however, since this is tedious and time-intensive, automatic segmentation procedures may be required to allow for a high-throughput image analysis. This approach of image processing might be challenging if images are taken under actual field conditions with soil as root growth medium and not under artificial conditions using nutrient solution or gel [11]. These shortcomings may be overcome by new machine learning techniques.

To recognize roots automatically, convolutional neural network (CNN)-based segmentation methods have been used recently, achieving a good performance [10, 12, 13]. Depending on the quality of a well-labelled training data set, machine learning can be applied on root images in actual soil environments which was so far adapted to analyze images of cotton and soybean [12, 13] and also wheat roots [10, 14, 15].

However, to our knowledge, the suitability of the combination of the minirhizotron approach with CNN-based neural network analysis for root measurement in wheat has not been shown so far. Previous studies either explored if a CNN may replace manual root segmentation in wheat root images [10] without reference to actual root data acquired by other means or used a CNN to reduce the workload in the analysis of destructively taken root samples [15], not referring to minirhizotron or similar images at all. [14], presenting the CNN RootPainter, used chicory data from a rhizobox experiment and also just compared the CNN approach to a manual image analysis. None of the mentioned studies and, to our knowledge, no other study compared minirhizotron measurements in the field and subsequent image analysis with the results from destructive root sampling. Our study therefore aims to critically evaluate the combination of the minirhizotron approach with CNN-based image analysis for the quantification of the root growth of winter wheat under field conditions.

For this purpose, we took minirhizotron images of wheat in three different crop rotational positions at several dates across the growing period in two study years in a long-term crop rotation trial. The objectives in detail were to (i) further develop and improve an existing CNN-based segmentation method for high-throughput image analysis of wheat minirhizotron images of wheat roots, (ii) verify the outcomes of the minirhizotron technique with root data from the destructive soil core method and (iii) investigate the effect of the presence of the minirhizotron tubes in the soil environment on root growth.

## Methods

### Field sites and experimental design

In this study, two field experiments were used in two vegetation periods (2021, 2022) each. The first field experiment was located near Harste (51°36’23.5"N, 9°51’55.8"E, 142 m above sea level) in Central Germany on a silty loam Luvisol derived from Loess [16]. The second field experiment was located at the Experimental Farm Hohenschulen (54°18′43.0′′N, 9°58′35.2′′E, 30 m above sea level) of Kiel University in Northern Germany on a sandy loam Luvisol [17]. Long-term (1991–2020) mean annual precipitation was 624 mm and 797 mm and mean annual temperature 9.4 °C and 9.3 °C at Harste and Hohenschulen, respectively [18].

The trial in Harste was established in 2006 [19] and compared nine crop rotations, out of which two with winter wheat were included in this study: (1) wheat monoculture (WM) and (2) winter oilseed rape (WOR)–winter wheat (WW)–winter wheat–grain pea–sugar beet–winter wheat. From the latter, the first (W1) and second wheat (W2) after WOR as break crop were considered. Each crop rotation element was cultivated every year, and each plot was replicated three times in a block design. Within plots, mineral N fertilization was varied as a second factor by applying 0 kg N ha^− 1^ (N_0_) and around 240 kg N ha^− 1^ (N_opt_), the latter to provide a total of 265 kg N ha^− 1^ including soil mineral N content in spring. For this, the main plot of 227 m² (14 × 16.2 m) was split up into two sub-plots resulting in a split-plot design with the crop rotational position on main-plot level and the N fertilization on sub-plot level.

The trial at Hohenschulen was established in 1989 [17] with one crop rotation: WOR-WW-WW-WW-faba bean-oats. The first (W1) and third (W3) wheat after WOR as break crop were considered. Each crop rotation element was cultivated every year. This trial contained no independent field replicates. Plot size was 54 m^2^ and N supply was 265 kg N ha^− 1^, including soil mineral N content in spring and N fertilization.

Before WW sowing in 2020 and 2021, reduced soil tillage was conducted with a cultivator to 12 cm soil depth in Harste, and to 15 cm after WOR and 25 cm after WW in Hohenschulen. Straw of the preceding crops was chopped during combining and incorporated by subsequent tillage. WW sowing density was 400 seeds m^− 2^ on 13 October 2020 and 18 October 2021 in Harste and 320 seeds m^− 2^ on 1 October 2020 and 8 October 2021 in Hohenschulen. The cultivar for both years and field sites was “Nordkap” (SAATEN-UNION GmbH, Isernhagen, Germany). Further crop management to adequately control weeds, pests and diseases followed the recommendations of the regional extension services adapted by the responsible technician.

## Destructive root sampling

Roots of winter wheat were sampled in both trials at growth stages BBCH 29 (mid to end of April) and BBCH 69 (mid to end of June) in 2021 and 2022 as shown in detail in [20]. Three soil cores per plot with a diameter of 60 mm were randomly taken in the wheat rows using a tractor-mounted hydraulic probe down to a depth of 120 cm and divided into depths of 0–15, 15–30 (combined to a composite 0–30 cm sample for the sake of this study), 30–60, 60–90 and 90–120 cm. Samples from 0 to 15 cm were not analyzed at the BBCH 69 sampling date, due to an exceedingly large amount of root and non-root organic material. For the sake of brevity, topsoil samples from both sampling dates, i.e. with the full 0–30 cm sampled and only the 15–30 cm depth sampled, are referred to as topsoil and 0–30 cm depth in the following.

Roots were washed out of the soil cores with water, manually cleaned from straw and plant residues and placed on a glass plate. Glass plates were scanned (Epson Perfection V850 Pro, Epson, Suwa, Japan) and analyzed for total root length with the software WinRHIZO 2019 (Regent Instruments, Quebec, Canada). Results for this analysis were presented and discussed by [20]. In this study, the data were used as reference for the accuracy of the minirhizotron results only.

## Minirhizotron installation and image acquisition

**Fig. 1:**
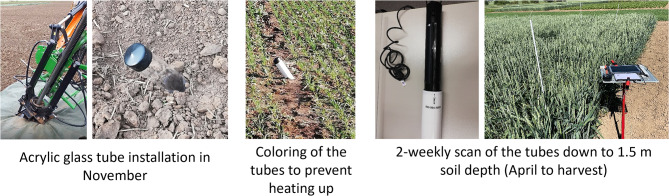
Overview of workflow of tube installation and tube scans

Field-installed acrylic glass tubes and the minirhizotron imaging system CI-600 (CI-600 In-Situ Root Imager, CID Bio-Science Inc., Camas, USA) were used for root growth observation during the vegetation period. In November 2020 and 2021, one month after wheat sowing, two (Hohenschulen) or three (Harste) tubes (2 m length and 70 mm outer diameter) per plot were installed in transverse direction to the seed rows at an angle of 30° from the soil surface (Fig. 1). To do so, first a hole with the outer diameter of the tube was drilled using a tractor-mounted hydraulic probe, afterwards the tube was manually inserted. Around 20 cm of the tube remained above ground and was colored first in black and on top in white to block out sunlight and avoid heating up the tubes and plant roots located next to the tube. Tubes were left in the field from installation until shortly before harvest when they had to be removed.

In 2022 in Harste, tubes were additionally slurried in with a mixture of soil obtained from the installation process and water to fix the tubes in the soil and avoid cavities around the tubes which were observed during 2021. Slurrying was done by manually inserting the soil-water mix with a watering can evenly around the tube. This was first done shortly after the installation and then repeated for two times with intervals of one week. The slurrying was not initially planned as treatment of the tubes to be tested, however, turned out to be a differentiating factor between the two study years due to the lack of experience with the method in 2021.

For measurements, the scanner CI-600 was inserted into the tube in overlapping depth intervals, capturing 360-degree RGB images of the tube-soil interface with an image resolution of 300 dpi and an image size of 21.6 × 19.6 cm. The scanner was connected to a handheld tablet and images were taken in up to seven soil depths along the tube to observe root development down to 120 cm vertical soil depth. Based on fixed scanner positions, image overlapping was 1 cm.

In Harste, images were captured biweekly from the beginning of April till the end of July in 2021 and 2022 resulting in seven to eight timepoints per year, while in Hohenschulen, images were taken at three timepoints across the growing period of 2022. The total number of images taken in Harste across both study years amounted to 6112 images while 400 images were taken in Hohenschulen. The Hohenschulen images were used as an independent dataset to test the performance of our approach on unknown images.

## Image processing - overview

**Fig. 2:**
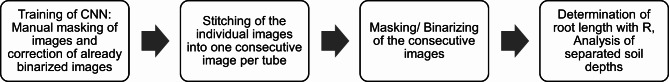
Overview of workflow of CNN adaptation and image processing

After initial training of the CNN, the experimental pipeline for root analysis commenced with the stitching, i.e. aligning and merging individual images of the tube to create one image per tube (Fig. 2). Subsequently, the segmentation step employed a CNN model to generate precise masks in black and white, isolating root structures from the background. In the final stage, individual parameter extractions utilized computational methods to quantify the root length. All steps are described in detail in the following.

## Infrastructure

The computational infrastructure consisted of an AMD Ryzen Threadripper PRO 3975WX processor with 32 cores clocked at 3.50 GHz, paired with 256 GB of RAM and an NVIDIA GeForce RTX 3080 GPU. Python (v3.9.0) and R (v4.2.3) served as the primary programming languages. Notable Python libraries encompassed CUDA Toolkit (v11.8.0), cuDNN (v8.9.2.26), Joblib (v1.2.0), Jupyter Notebook (v1.0.0), Matplotlib (v3.7.1), NumPy (v1.24.2), OpenCV-Python (v4.7.0.72), Pillow (v9.4.0), scikit-image (v0.20.0), scikit-learn (v1.2.2), SciPy (v1.9.1), PyTorch (v1.13.1 + cu116), among others R (v4.2.3), executed via RStudio (v2023.03.0 + 386), data.table (v1.14.8), dplyr (v1.1.1), EBImage (v4.40.1), ggplot2 (v3.4.2), RCurl (v1.98-1.12), readxl (v1.4.2), stringr (v1.5.0), tidyr (v1.3.0) and zoo (v1.18-11).

### Stitching

**Fig. 3:**
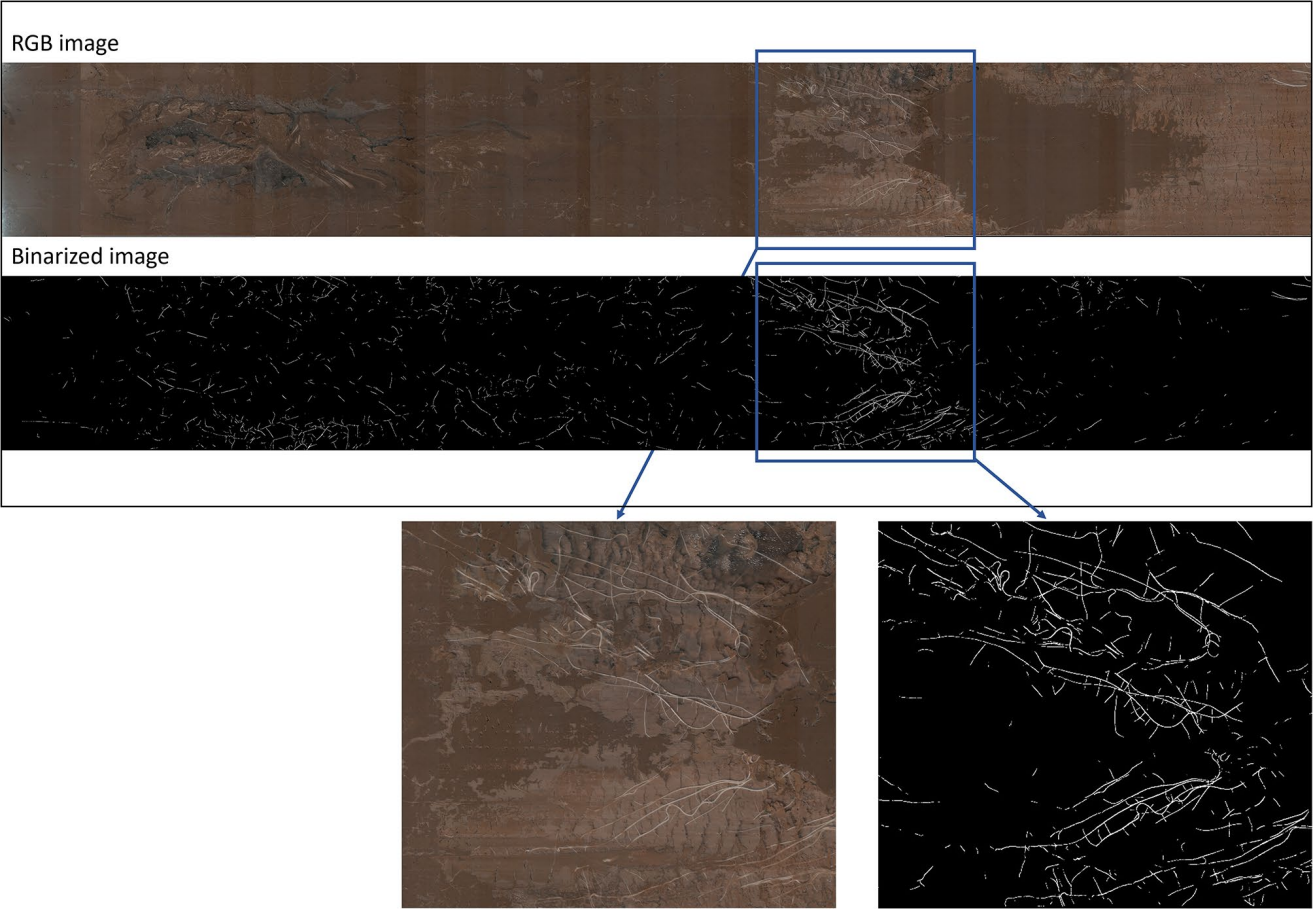
Stitched RGB image of minirhizotron scan and binarized image based on CNN with train dice of 0.72. RGB image was taken in first wheat after oilseed rape (W1) with no N fertilization (N_0_) on 23rd May 2022 in Harste. Left side of images = top of tube, right side of images = bottom of tube

The consecutive single images were stitched to a complete image per tube and segmented from the background with the improved SegRoot [13]. Figure 3 gives a detailed example of the stitching and segmentation result with the improved SegRoot [13]. The stitching process presented a significant challenge due to the minimal overlap between consecutive images and the high homogeneity of their backgrounds. To address this, we employed the Sci-images tools in Python and followed a systematic approach.

First, in the preprocessing stage, we converted the color images to grayscale using the rgb2gray function. To enhance the final stitched result and reduce redundancy, we selectively removed overlapping regions by zeroing out pixel values in both the left and right images. The degree of overlap removal was parameterized to retain a specified percentage of the right image. Next, we utilized the ORB feature detector and descriptor to identify key points and generate descriptors for both images. ORB is an efficient feature detector and descriptor that combines the FAST key point detector and the BRIEF descriptor [21]. The FAST key point detection algorithm identifies points of interest in an image by comparing the intensity of a pixel with its surrounding pixels. It is known for its speed and simplicity. On the other hand, the BRIEF descriptor creates a compact and efficient representation of local image regions using simple binary tests between pairs of pixels. ORB introduces a learning method to de-correlate BRIEF features under rotational invariance and enhances FAST by adding a fast and accurate orientation component [21].

The match_descriptors function from the skimage.feature module [22] compares each descriptor from one set to all descriptors in another set using Hamming distance. This comparison identifies and ranks potential matches based on similarity, optionally enforcing a cross-check to ensure bidirectional consistency, thereby establishing reliable correspondences between key points from different images. To robustly estimate a projective transformation model, we employed the Random Sample Consensus (RANSAC) algorithm. This algorithm allowed to identify inliers, which were crucial for fitting a reliable transformation model while mitigating the impact of outliers. Based on the inliers obtained through RANSAC, we performed cropping on the images, excluding the overlapping regions. This step ensured that the final stitched result comprised only non-overlapping portions from each input image. Finally, the generation of the ultimate stitched image (combined_image) involved the horizontal concatenation of the cropped left and right images. This meticulous process resulted in a seamless and accurate combination of the input images, overcoming the challenges posed by limited overlap and background homogeneity.

## Root segmentation

The consecutive images of each tube were processed with a modified version of SegRoot, a fully automated method based on CNNs and adapted for segmenting roots from complex soil [13]. SegRoot was originally designed for image segmentation of soybean root images for which the testing dice score was 0.6441. Dice coefficient or score [23] is a measure of the similarity between two sets and is calculated as:

$$\eqalign{& {\rm{Dice}}{\mkern 1mu} \ {\rm{score}}{\mkern 1mu} \,{\rm{ = }}  & \,\,\,{{ {\rm{Ground}}{\mkern 1mu}  \ {\rm{Truth}}{\mkern 1mu} \ {\rm{Positives + }}{\mkern 1mu} {\rm{Predicted}}{\mkern 1mu} \ {\rm{Positives}}} \over {{\rm{2}} \times {\rm{True}}{\mkern 1mu} \ {\rm{Predicted}}{\mkern 1mu} \ {\rm{Positives}}}} \cr}$$

where:

Ground Truth Positives: The number of positive pixels or elements in the ground truth.

Predicted Positives: The number of predicted positive pixels or elements.

True Predicted Positives: The number of correctly predicted pixels or elements referring to ground truth.

Dice expresses the balance between precision and recall in binary classification tasks, providing a value between 0 and 1, where 1 indicates perfect agreement between the sets. When predicting roots with the original SegRoot procedure in our image dataset, we observed average to bad performance (dice 0.58), which could be attributed to several factors. Notably, variations in background, diverse camera specifications, and the presence of different types of roots likely contributed to the deviation. Moreover, our images were captured under real field conditions, including complexities such as adverse weather conditions and residues from other plants and previous years. These environmental factors can significantly impact the robustness and consistency of image analysis results.

The code published by [13] was in parts rewritten and adapted to use PyTorch = < 2 since the original was trained on PyTorch 0.4.0 and many of the functions have been modified or relocated. The model architecture was slightly modified to be less susceptible to overfitting by adding dropout in during the encoder and decoder layer (0.2). It was also applied during forward L2 regularization with a dropout weight of 0.0001. Further, we added an early stopping to the model to avoid overfitting, saving time and resources by stopping the training when performance on validation data stops improving significantly, in our case when the validation loss did not improve for 20 epochs.

During the binarization of the images, the images were cut into 256 patches. Here an increase in data for training was included by performing a 30% overlap at the time of cutting. After that, a series of data augmentation transformations were performed on each image and its corresponding mask. These transformations include (i) dilate or morphological close, (ii) rotation, (iii) zoom, (iv) horizontal and vertical flip, (v) brightness and contrast adjustment and (vi) hue and saturation adjustment. At a dilate or morphological close a random choice is made between “dilate” and “close” as operation and applied to the mask. The dilate increases the size of the white pixel regions, while the close reduces the holes in the white pixel regions. During the rotation the images and masks were rotated randomly by an angle of 0, 90, 180 or 270 degrees, while during zoom a random zoom factor between the range of 0.9 and 1.1 was applied to images and masks. Images and masks were scaled to make them slightly larger or smaller. The horizontal and vertical flip added spatial variability by performing randomly a horizontal and/or vertical flip to images and masks. The brightness and contrast of the images was randomly adjusted in the range of 0.9 to 1.1. In addition, the hue and saturation of the images in the HSV color space were randomly adjusted. The hue factor was randomly chosen in the range of -0.1 to 0.1, while the saturation factor was randomly chosen in the range of 0.9 to 1.1.

Using our image dataset, we initially applied the original SegRoot model. Then, we carefully went through the process of adjusting and enhancing 204 individual images. We next used this subset to fine-tune our model in order to improve its performance and better match it to the subtleties of our dataset.

Once the model was trained and selected, it was applied to our images to predict the binary masks of each stitched image.

## Feature extraction

A series of image processing operations were executed, adapting the workflow proposed by [24], initially designed for automated histological assessment, for the segmentation of individual roots using watershed segmentation [25, 26] and precise root length measurement. Following the initial steps of the [24] methodology, the root mask image (binary mask) was loaded, and morphological operations including interluding, noise reduction, and opening were applied. Subsequently, watershed segmentation was employed on the labeled components of the dilated mask, resulting in a segmented image. Connected components in this segmented image were labeled, and shape features were computed to ascertain the root length of each segmented root group. The root length was further categorized based on different vertical soil depths, specifically 0–30 cm and 30–120 cm increments, mirroring the depth increments used for destructive root samples. In addition, root length was calculated for the vertical soil depth increments 30–60, 60–90 and 90–120 cm for the study year 2022 for the minirhizotron data as well as for the destructive root samples. The conversion of the soil depth along the minirhizotron into a vertical soil depth was carried out using the Pythagorean theorem, the angle sum of the triangle of 180 degrees and the sine function to calculate the missing opposite catheter. The missing angle is 60 degrees, as the plexiglass tube was installed at an angle of 30 degrees and the vertical soil depth is at an angle of 90 degrees to the soil surface. The following formula was used to calculate the length:

1 $$soildepth\_plexiglasstube = {\rm{ }}t{\rm{ }} \ cm/{\rm{ }}sin\left( {60^\circ } \right)$$

with t = 15 or 30 cm depending on the vertical soil depth to compare.

To evaluate a potential bias effect of the presence of the tube on root growth, differences in root length between the four quarter sections (above, below, left, right) around the tube were analyzed separately for both study years. The background for this analysis was the assumption that if the tube has some influence on root growth this would be shown as preferential growth along the tube and not downwards as usually to be expected, and that this growth along the tube should be more visible at the downside of the tube since this would be where the root would grow away from the tube again, if there were no effects of the tube presence. That way, also the potentially ameliorating effect of slurrying the tubes could be tested since slurrying was only done in 2022.

### Statistical analyses

The statistical data analysis was conducted with R version 4.2.3 (R Foundation for Statistical Computing, Vienna, Austria). Differences in root length between the quarters around the tubes were analyzed separately for each study year by a linear mixed model ANOVA with quarter around tube (above, below, left, right), scan date and its interaction, as well as replication as fixed effects, and plot, subplot, and the interaction between tube, plot and replication as random effect.

The linear mixed model was calculated with package “lmerTest” (v3.1-3). The residuals of the models were checked for normal distribution graphically and with Shapiro-Wilk’s test, and for homoscedasticity with the Levene’s test as well as graphically. No interaction was determined between quarter around tube and scan date. If the factor quarter around the tube was significant (*p* < 0.05), means were compared by a post-hoc Tukey test with the package “emmeans” (v1.8.5). The relationship between root length density measured with the destructive core method and root length measured with the minirhizotron technique was further analyzed with a linear regression model calculated with SigmaPlot (version 14.5, Systat Software Inc., USA) for the top- and subsoil (0-30 and 30-120 cm, respectively) in both years at Harste, as well as in one study year at Hohenschulen. Additionally, a more detailed analysis was conducted for the three subsoil depths (30-60, 60-90 and 90-120 cm) in 2022 in Harste.

## Results

Our adapted CNN was rigorously trained over the course of 405 epochs. Upon completion of this training regimen, the model demonstrated a testing dice score of 0.7203, signifying a robust capability in accurately segmenting the test dataset. The training process yielded a dice score of 0.6637 on the training dataset, illustrating the model’s learning progression and adaptation. Additionally, the model achieved a validation dice score of 0.6929, indicating a strong generalization performance on the validation dataset.

**Fig. 4:**
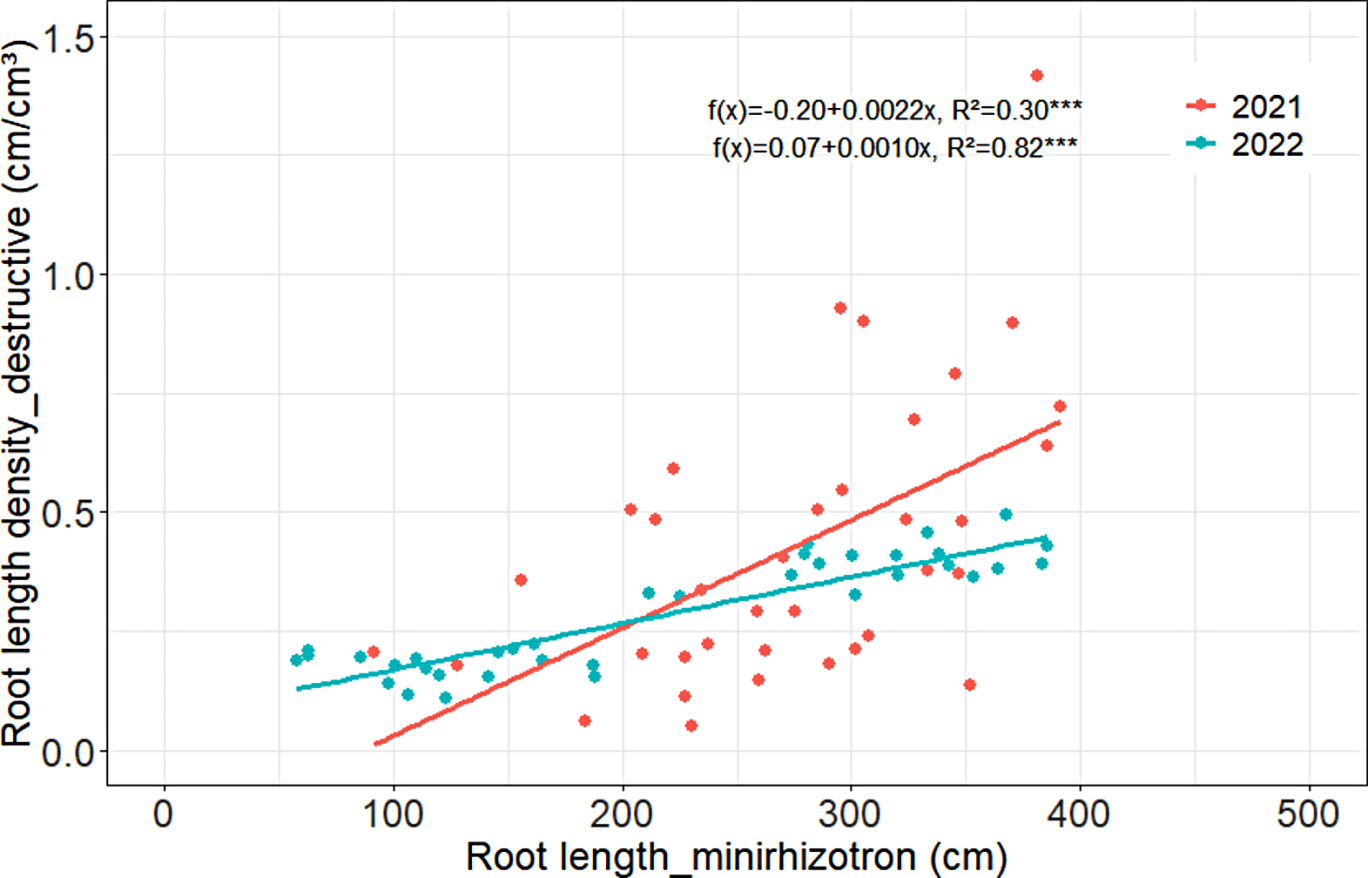
Relationship between root length density of winter wheat measured by destructive soil coring and root length obtained by minirhizotron technique in 30–120 cm soil depth in Harste, data from 2021 and 2022, *n* = 36. Asterisks indicate significant coefficient of determination at *p* < 0.001***

**Fig. 5:**
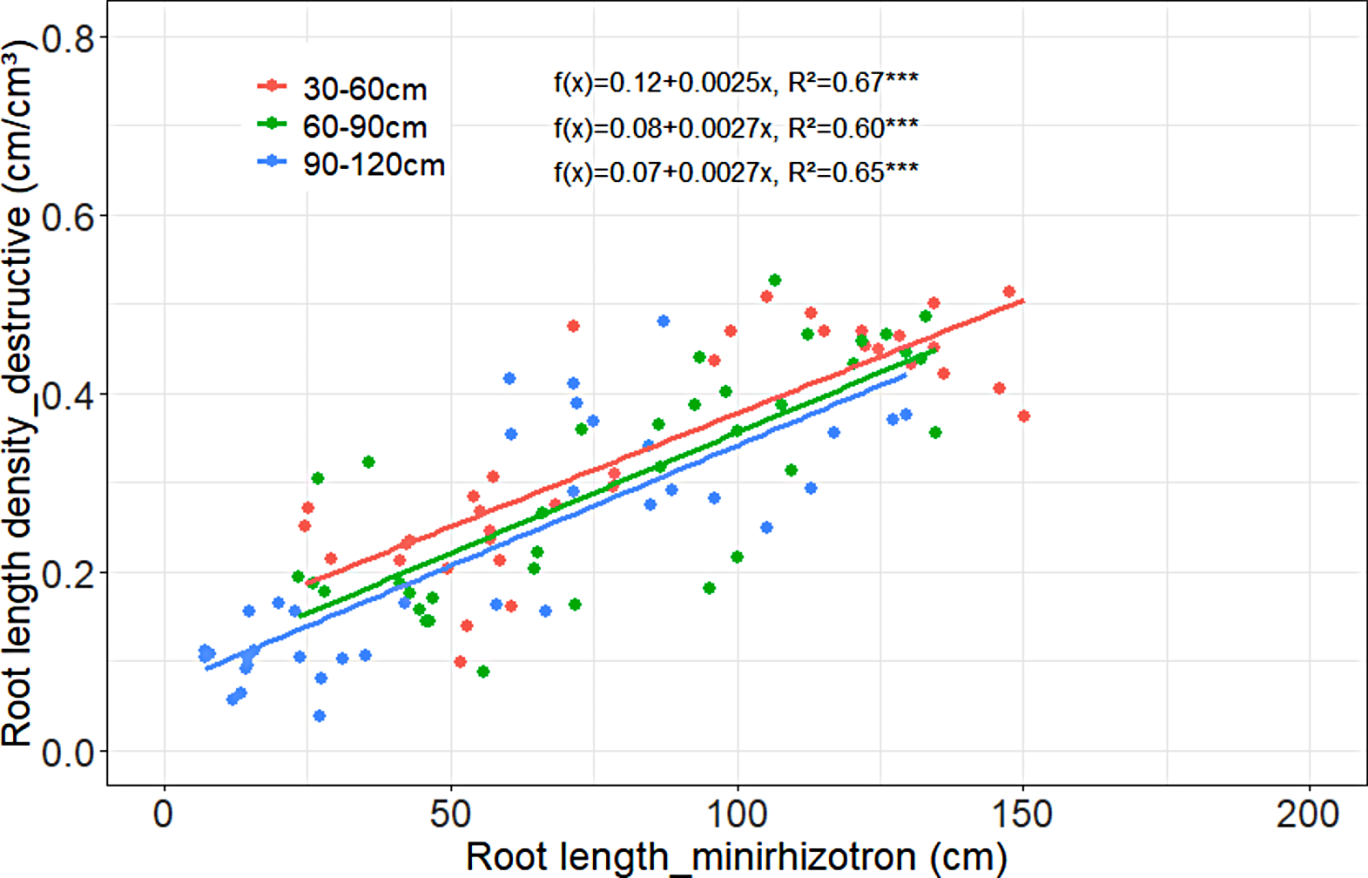
Relationship between root length density of winter wheat measured by destructive soil coring and root length obtained by minirhizotron technique in different soil depths in Harste in 2022, n_single_depth_ = 36. Asterisks indicate significant coefficient of determination at *p* < 0.001***

In Harste in 0–30 cm soil depth, no significant relationship between the destructively measured root length density and root length measured with minirhizotrons could be determined in both study years (2021: f(x) = 1.12 + 0.0044, R²=0.01, 2022: f(x) = 0.52 + 0.0007, R²=0.06). In 30–120 cm soil depth, however, the regression analysis of the 2021 data showed a low, yet significant, positive relationship between the root length density measured destructively compared to the root length measured with minirhizotrons, whereas in 2022 a strong positive relationship was found (Fig. 4). When further dissecting the 2022 subsoil data into 30–60, 60–90 and 90–120 cm soil layers, the regression analysis consistently revealed a close relationship in each soil layer (Fig. 5) with R² values between 0.60 and 0.67.

**Fig. 6:**
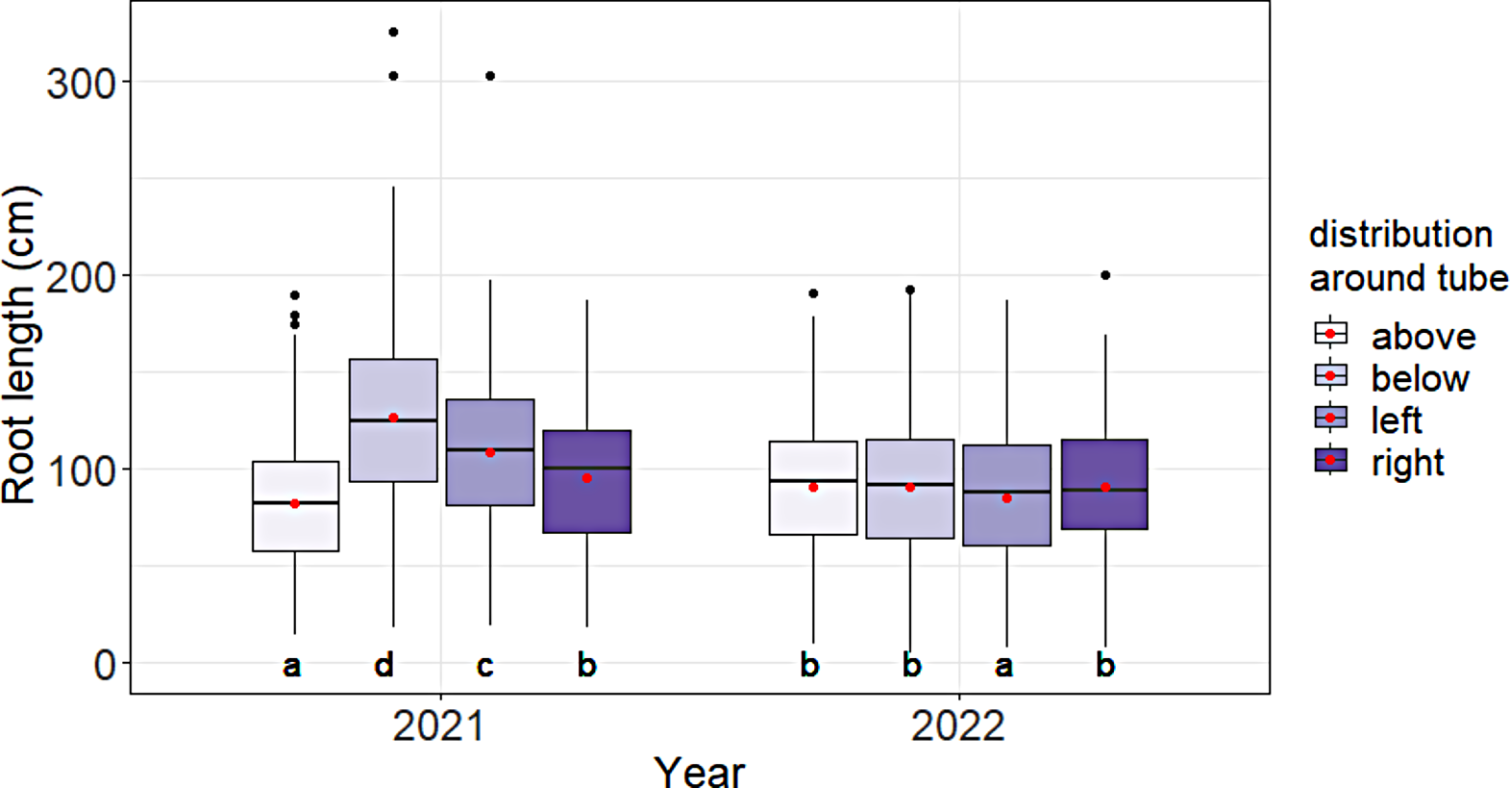
Root length distribution around the minirhizotron tube in Harste, data from 2021 (*n* = 378) and 2022 (*n* = 432). Black lines in boxplots show median and red dots show mean. Lowercase letters show significant differences between means in each area around tube within one study year (*p* < 0.05)

In 2021, without slurrying of the tubes, the root length was significantly higher below the tube (126.4 ± 48 cm) than on the left side (108.2 ± 40 cm), the right side (94.7 ± 35 cm) or above the tube (81.5 ± 33 cm) (Fig. 6), while in 2022, with slurrying of the tubes, differences were low and not significant, with values ranging between 85.1 ± 34 cm and 90.4 ± 35 cm.

When the area below the tube was excluded from the analysis for 2021, the regression analysis for root length density measured destructively and root length measured via the minirhizotrons for the 30–120 cm soil depth in 2021 also revealed a strong significant relationship with f(x) = 0.10 + 0.0104x and R²=0.57***.

The scan images from the field trial Hohenschulen were included in this study as a dataset which was not used for the previous training. Visual examination of the segmented images revealed no performance degradation of the approach compared to the images used for training, and the regression analysis showed a significant relationship between the root length measured destructively and the root length measured with minirhizotrons in both 0–30 and 30–120 cm soil depth (Supplementary Fig. 1).

## Discussion

### Suitability of the CNN approach for wheat root detection in images

The high testing dice score of 0.7203 after 405 epochs of the developed CNN confirms the suitability of the CNN used to segment winter wheat roots from the field soil background in the used datasets of RGB images from minirhizotrons. Compared to the testing dice score of 0.6441 by [13] we achieved a higher score and could thus improve the root segmentation with SegRoot. The good performance was confirmed by the application to unknown images from the trial in Hohenschulen. This fits to results from [13], who observed no distinguishable performance degradation of their CNN SegRoot on never seen images of soybean and tree roots. A well-trained CNN can learn to recognize root patterns that are invariant to changes in the background, including different soil types. Further, it is capable to capture contextual information from the surrounding pixels, which can help in distinguishing between different soil types. To achieve this, however, it is important to have a high number of high resolution and sufficiently heterogeneous datapoints for training [27]. For a well-developed and trained CNN, different soil backgrounds or soil types should therefore not be an obstacle for image segmentation. Additionally, since we used an already existing CNN, trained on other data including different soil conditions, and adapted it, more than only the Harste soil type is already present in the CNN.

Generally, a CNN was recommended as the best method to segment plant organs from a specific background [28]. With our results we can confirm the suitability of the CNN approach for this use. The established workflow allows for a high-throughput image analysis of RGB images of winter wheat roots.

### Comparison of soil core root sampling and minirhizotron technique

For both study years in Harste no relationship between soil core root sampling and minirhizotron measurements could be determined for the 0–30 cm soil depth, while a significant relationship was found for Hohenschulen 2022. However, the minirhizotron data in 0–30 cm showed only little variation at all, while the destructively measured data, at least in Harste 2021, varied strongly (data not shown). Overall, the root length in 0–30 cm as measured by the minirhizotron approach was very low across the study sites (maximum around 100 cm), particularly compared to the 30–60, 60–90 and 90–120 cm soil depths (up to 150 cm root length), for which a generally lower root length should have been expected. Despite the significant relationship between the minirhizotron and the soil core data in Hohenschulen, yet supported by the lack thereof in Harste, this might indicate that the minirhizotron technique is not suitable for the measurement of root length in the topsoil. This could be due to physical disturbances through the tubes during installation, causing non-representative root and overall plant growth of the surrounding wheat plants. Similarly, [29] reported problems when comparing both techniques in 0–30 cm soil depth and stated that when tubes were installed after growth had started, roots already present at that time were destroyed and a good soil-tube contact was hard to secure in a small timeframe afterwards. [30, 31] even reported problems when the tubes were installed immediately after sowing which was also the case in our study. [32] concluded that the minirhizotron technique must be calibrated with other root growth measurement techniques and that this is necessary especially in the topsoil. Thus, topsoil root data measured with the minirhizotron technique might not be reliable and meaningful.

Roots detected in the subsoil, in contrast, originate from plants further away from the point of installation and might thus have been less disturbed by installation in autumn. The latter assumption is apparently confirmed by the strong relationships between the root length density measured destructively and the root length obtained from minirhizotrons in 30–120 cm soil depth as well as subdivided in 30–60, 60–90 and 90–120 cm soil depth in 2022, while in 2021 the relationship between both methods was lower. This might have been caused by the missing slurrying of the minirhizotron tubes, which was done in 2022 to assure a good soil-tube fitting avoiding voids around the tubes but was not done in 2021 due to a lack of experience with the method. [33] also reported that a good soil-tube contact along the tube surface is essential for a meaningful root observation. Similarly, for the Hohenschulen trial, no slurrying took place, which may responsible for the comparably low (R² = 0.34) relationship of the minirhizotron data with the soil core data, as also found in Harste 2021. Small-scale texture variability, which is substantially higher at the Hohenschulen site, is another possible reason for the lower consistency between the methods.

The lack of a sufficiently close contact between tube and soil also likely led to the heterogeneous distribution of roots around the tubes in 2021, with a higher root length below the tube compared to the other sides. The missing slurrying might have caused large voids, which, particularly below the tube, were probably not closed by soil settlement over winter. Such holes might then have promoted wheat root growth similarly to large biopores (shown for biopores by e.g [34–36]), causing a high root length. Similarly, [32, 37] and [38] reported that large holes around the minirhizotron tubes created by the installation procedure can cause an abnormally large root growth, which was especially observed in the subsoil by [38], while in the topsoil a good soil-tube contact was achieved. In our study, in 2022, all tubes were slurried and an even distribution of the roots around the tubes was found. With the possibly artificially created biopores in 2021 in mind, the area below the tube was excluded from the dataset which led to a much stronger relationship between the root length measured via the two methods in 2021, identifying this part of the measurements as indeed probably problematic. These findings lead to the strong recommendation to secure a close tube-soil contact in order to minimize the risk of biased data.

Regarding the workload for the two methods, installation of one tube took approximately as long as taking one destructive root core sample. Since the number of installed tubes and the number of root samples were identical per plot (three), the time needed for the initial field work was thus similar for both approaches. With a well-established workflow for the image analysis, the subsequent image processing and final analysis, however, can be done by one person in several hours to at most a few days per sampling date (in our case 54 tubes down to at least 1.2 m), while the manual analysis of one sampling date of destructive root samples (54 samples x 5 soil depths) might consume several months if one person were to do the analysis alone (in our experience, an average of five samples per working day can be expected). Thus, the minirhizotron technique may be a valid and substantially less work-intensive alternative for the highly labor-intensive destructive soil core sampling.

As another aspect, however, the costs for both methods need to be considered. In our case, installation of the tubes and sampling of the roots was done with the same device specifically manufactured for these purposes, costing a medium five-figure amount (EUR currency). While root samples can be taken by other, less costly means, tube installation to a certain depth at an angle of 30° is a very demanding task for the machinery used and could otherwise only be done manually, which would greatly increase the labor needed for installation. Also, of course, the scanner itself and the tubes have to be purchased, resulting in a low five-figure amount to be spent. For the root samples, further costs have to be noted, for the high-quality tubular film needed for each sampling spot, glass plates, a scanner of sufficient quality and the software WinRhizo needed for the subsequent analysis of the scans. All these additional costs in mind, the material costs are probably higher for the minirhizotron approach, however, these costs are mostly connected to the first purchase of the system while taking destructive samples causes ongoing costs, primarily for labor. Considering these labor costs, the final appraisal in terms of costliness will likely be favorable for the minirhizotron technique.

### Possible limitations of the minirhizotron technique

Limitations of the minirhizotron technique might be observed in dry periods, as seen in our dataset at the penultimate scan date in 2022. The weather data for that week showed an absence of precipitation followed by two weeks with high precipitation (Supplementary Fig. 2). A detailed look on several images of the two scan dates, exemplarily shown in Supplementary Fig. 3, revealed that missing precipitation caused shrinking of the soil, thereby loosening and offsetting the soil from the tube surface, even after a good soil-tube contact was established before via slurrying. In consequence, the CNN was not able to segment roots correctly. After a period with high precipitation the scan images showed a re-swelling of the soil; subsequently the CNN was again able to segment all roots from the soil background. This is another confirmation that a good soil-tube contact is essential for meaningful root growth observation [33].

In general, the unavoidable limitation of insufficient root detection in severe drought conditions might limit the usability of minirhizotrons in arid and semi-arid environments where these conditions have to be expected regularly. In humid conditions, as in our study, a critical evaluation of the weather and soil conditions during measurement certainly is advisable alongside minirhizotron measurements to ensure no misleading results are produced. However, since we would only recommend using minirhizotrons for subsoil measurements and given that the “dry” scan date in 2022 was in a particularly dry period within an exceptionally dry year, i.e. a rather extreme example, we would not expect these circumstances to occur frequently.

Moreover, [13] reported that SegRoot is biased in detecting blurred areas and water condensation as false negative which was already discussed by [39] as early as 1990. This issue was improved by our model modifications, as no misleading segmentation due to blurriness or water drops was noticed by a human control and also because we incorporated images with this problem into our data set. In addition, slurrying the tubes may help to reduce the number of waterdrops on the images so that this issue does not come up at all. This likely enhanced the clarity of the images and further improved the accuracy of root detection.

Also, adaptation and fine-tuning of the applied CNN may be required to optimize image analysis performance on new soils since the soil background of our training dataset was not strongly varying. However, using data augmentation, as done in this study, and relying on the high transferability of a well-trained CNN to different environments, we would expect a good performance on other soils as well.

Still, there might be environmental situations where minirhizotron measurements are not advisable due to weather conditions (see also above) or also soil conditions potentially disturbing measurements that we currently do not think of. Under these kinds of circumstances, soil coring might still be feasible and thus the method of choice. However, we assume that under a large variety of environmental conditions, minirhizotrons may add information and, depending on the research question, may indeed possibly replace soil coring if the required technology and knowledge is available and/or if the needed workforce for soil coring and subsequent root analysis is not.

## Conclusions

The minirhizotron technique in combination with subsequent high-throughput image analysis via CNN was found to allow for less labor-intensive yet potentially similarly precise root length measurements during the whole vegetation period in comparison to destructive soil core sampling. This particularly refers to the subsoil, while in the topsoil results were dissatisfactory. However, since the subsoil may be of greater interest and is harder to reach with destructively taken soil cores than the topsoil, minirhizotrons may be a valuable technique in root research, potentially supplementing or even replacing deep soil coring if the tubes are sufficiently slurried after installation. This approach could also be beneficial for root phenotyping of different wheat genotypes. Further investigations are needed to examine if the CNN developed for the segmentation of winter wheat roots can also be used for images of other crops with further adaption. Also, since most studies focusing on tube installation issues seem to be rather old, more methodological studies using currently available systems are needed to identify potential pitfalls in the field beyond the ones described in this study.

## Electronic Supplementary Material

Below is the link to the electronic supplementary material.

Supplementary Material 1: Supplementary Fig. 1 Relationship between root length density of winter wheat measured by destructive soil coring and root length obtained by minirhizotron technique in different soil depths in Hohenschulen, data from 2022, n_0 − 30 cm_ = 15, n_30 − 120 cm_ = 16. Asterisks indicate significant coefficient of determination at *p* < 0.05 and *p* < 0.001***. Supplementary Fig. 2 Weekly mean air temperature (°C, black line) and sum of precipitation (mm, grey bars) for the study years 2021 and 2022 in Harste. Black vertical line = separation of years; grey areas = vegetation periods with mean air temperature and precipitation sum; dark grey area = period with soil loosening on minirhizotron tubes, numbers = precipitation sum > 35 mm. Supplementary Fig. 3 Effect of soil shrinking and swelling caused by drying and rewetting on visibility of roots on RGB images of minirhizotron scans at 05th July 2022 and 18th July 2022 in Harste. Red lines show area of loosened soil due to lack of precipitation. Top = top of tube, Bottom = bottom of tube.

## Data Availability

The datasets used analyzed during the current study are available from the corresponding author on reasonable request.
