# Correction: Minirhizotron measurements can supplement deep soil coring to evaluate root growth of winter wheat when certain pitfalls are avoided

**DOI:** 10.1186/s13007-025-01328-1

**Published:** 2025-01-27

**Authors:** Jessica Arnhold, Facundo R. Ispizua Yamati, Henning Kage, Anne-Katrin Mahlein, Heinz-Josef Koch, Dennis Grunwald

**Affiliations:** 1 Institute of Sugar Beet Research, Holtenser Landstraße 77, 37079 Göttingen, Germany; 2 Institute of Crop Science and Plant Breeding, Kiel University, 24118 Kiel, Germany

**Correction:** **Plant Methods (2024) 20:183** 10.1186/s13007-024-01313-0

In this article [1] Facundo R. Ispizua Yamati and Jessica Arnhold should have been denoted as an equally contributing author.
