# Supplementary material for: Minirhizotron measurements can supplement deep soil coring to evaluate root growth of winter wheat when certain pitfalls are avoided
Source: Plant Methods. 2024 Dec 17;20:183. doi: 10.1186/s13007-024-01313-0 (PMC11650824; doi:10.1186/s13007-024-01313-0)
Supplement: Supplementary file 1 — Supplementary Material 1: Supplementary Fig. 1 Relationship between root length density of winter wheat measured by destructive soil coring and root length obtained by minirhizotron technique in different soil depths in Hohenschulen, data from 2022, n0 − 30 cm = 15, n30 − 120 cm = 16. Asterisks indicate significant coefficient of determination at p < 0.05 and p < 0.001***. Supplementary Fig. 2 Weekly mean air temperature (°C, black line) and sum of precipitation (mm, grey bars) for the study years 2021 and 2022 in Harste. Black vertical line = separation of years; grey areas = vegetation periods with mean air temperature and precipitation sum; dark grey area = period with soil loosening on minirhizotron tubes, numbers = precipitation sum > 35 mm. Supplementary Fig. 3 Effect of soil shrinking and swelling caused by drying and rewetting on visibility of roots on RGB images of minirhizotron scans at 05th July 2022 and 18th July 2022 in Harste. Red lines show area of loosened soil due to lack of precipitation. Top = top of tube, Bottom = bottom of tube. [file 13007_2024_1313_MOESM1_ESM.pdf]

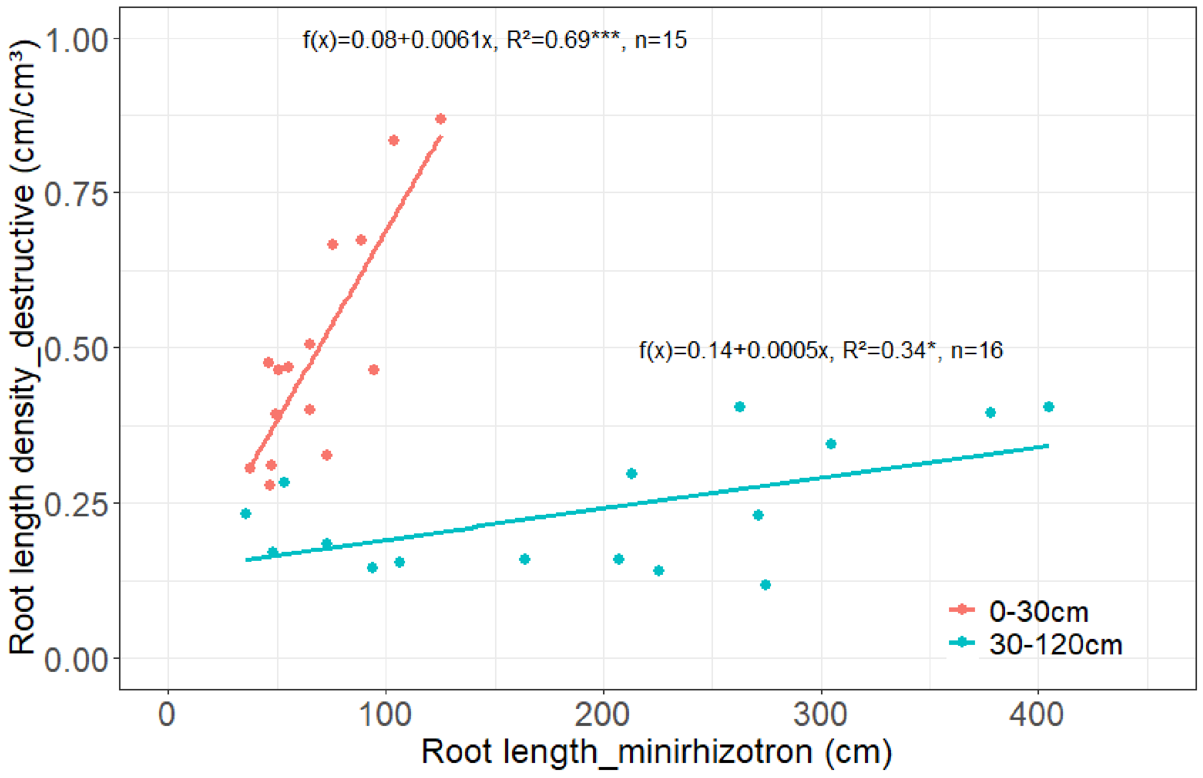

Supplementary Figure 1: Relationship between root length density of winter wheat measured by destructive soil coring and root length obtained by minirhizotron technique in different soil depths in Hohenschulen, data from 2022, $n_{0\text{-}30cm}$ = 15, $n_{30\text{-}120cm}$ = 16 . Asterisks indicate significant coefficient of determination at p< 0.05 and p<0.001***.

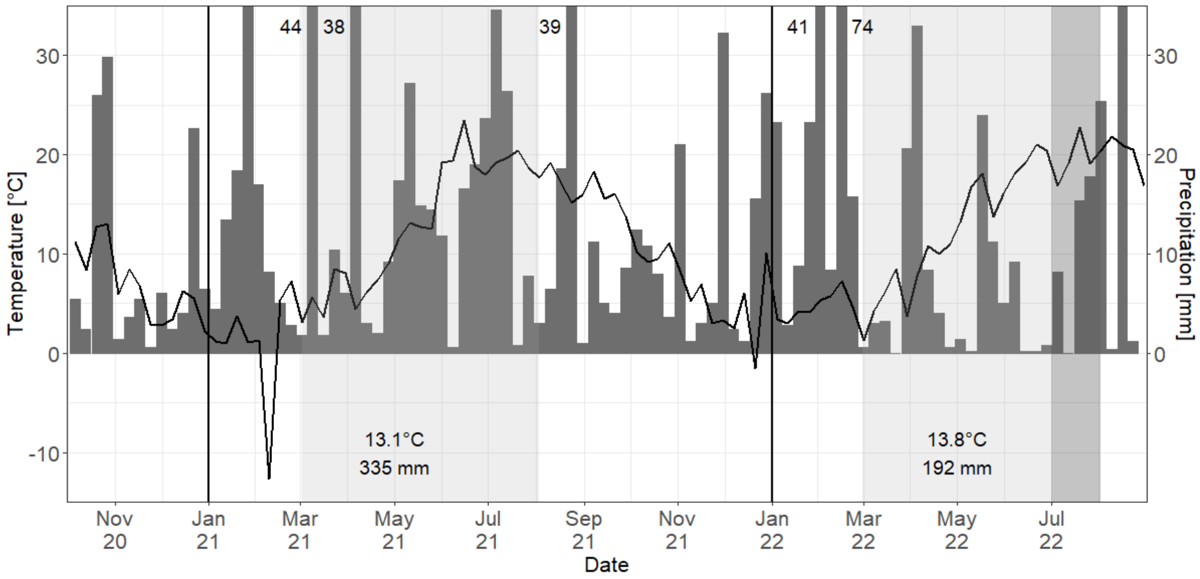

Supplementary Figure 2: Weekly mean air temperature (°C, black line) and sum of precipitation (mm, grey bars) for the study years 2021 and 2022 in Harste. Black vertical line = separation of years; grey areas = vegetation periods with mean air temperature and precipitation sum; dark grey area = period with soil loosening on minirhizotron tubes, numbers = precipitation sum > 35 mm.

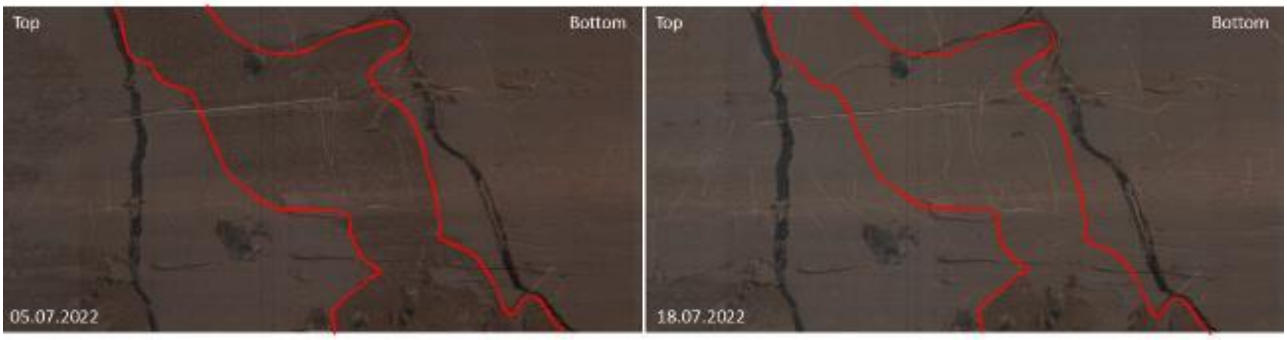

Supplementary Figure 3: Effect of soil shrinking and swelling caused by drying and rewetting on visibility of roots on RGB images of minirhizotron scans at 05th July 2022 and 18th July 2022 in Harste. Red lines show area of loosened soil due to lack of precipitation. Top = top of tube, Bottom = bottom of tube.
